# Assessing the Efficacy of Modified CT Severity Index Versus Conventional CT Severity Index in Determining Severity and Clinical Outcomes of Chronic Obstructive Pulmonary Disease (COPD)

**DOI:** 10.7759/cureus.73771

**Published:** 2024-11-15

**Authors:** Ali Ashraf Cheema, Gul Sharif, Sanjay Kirshan Kumar, Hamza Naseer Butt, Ibrahim Ali Khan, Jawad Hussain, Hiba Manzoor, Nawaf Safaq Alshammari, Fauwaz F Alrashid

**Affiliations:** 1 Emergency Medicine, Gondal Hospital Gujranwala, Gujranwala, PAK; 2 Surgery, Lady Reading Hospital, Peshawar, PAK; 3 Gastroenterolgy, Bahria University Medical and Dental College, Karachi, PAK; 4 Acute and General Internal Medicine, Queen Elizabeth University Hospital, Glasgow, GBR; 5 Pulmonary Medicine, Darul Sehat Hospital, Karachi, PAK; 6 Medicine, Sahara Medical College, Narowal, PAK; 7 Internal Medicine, Lahore Medical and Dental College, Lahore, PAK; 8 Family Medicine, King Abdul-Aziz Medical City, National Guard Health Affairs, Riyadh, SAU; 9 Surgery, College of Medicine, University of Hail, Hail, SAU

**Keywords:** chronic obstructive pulmonary disease (copd), clinical outcomes, computed tomography severity index (ctsi), disease severity assessment, modified ct severity index (mctsi)

## Abstract

Aims

The aim of this study was to evaluate the severity of chronic obstructive pulmonary disease (COPD) using the computed tomography severity index (CTSI) and the modified CTSI (MCTSI) and to assess their correlation with clinical outcome measures. Additionally, the study aimed to compare the diagnostic performance of these indices in predicting moderate to severe COPD, based on patient outcomes.

Materials and methods

In this prospective study, conducted between November 2023 and March 2024, two radiologists, blinded to clinical outcomes, independently assessed CTSI and MCTSI. Clinical outcomes evaluated included the duration of hospital stay, intensive care unit (ICU) stay, organ failure (OF), evidence of infection, need for intervention, and mortality.

Results

The study included 60 COPD patients, with a majority being male (40, 66.7%) and a mean age of 65.4 ± 8.6 years. Based on CTSI, severity was classified as mild in 25 (41.7%), moderate in 20 (33.3%), and severe in 15 (25.0%) cases. According to MCTSI, severity was classified as mild in 22 (36.7%), moderate in 15 (25.0%), and severe in 23 (38.3%) cases. MCTSI showed concordance with the Global Initiative for Chronic Obstructive Lung Disease (GOLD) classification in 54 (90.0%) cases, while CTSI was concordant in 47 (78.3%) cases. For predicting moderate to severe COPD, CTSI demonstrated a sensitivity of 91.4%, specificity of 96.0%, positive predictive value (PPV) of 97.1%, and overall accuracy of 98.3%. MCTSI showed a sensitivity of 94.4%, specificity of 92.0%, PPV of 94.4%, and accuracy of 96.7%. Both indices correlated significantly with clinical outcomes, including OF, need for intervention, infection, and mortality (p < 0.001), although no significant correlation was found with ICU stay.

Conclusion

Both CTSI and MCTSI demonstrated a strong correlation with clinical outcomes in COPD patients and showed good concordance with severity assessments. MCTSI exhibited higher sensitivity, while CTSI demonstrated greater specificity for differentiating mild from moderate/severe COPD cases. These indices can be valuable tools in guiding clinical decision-making and assessing disease severity in COPD.

## Introduction

The primary symptoms of chronic obstructive pulmonary disease (COPD), a complex disorder primarily brought on by inhalational pollutants, negatively impact the lungs [1]. COPD is expected to overtake all other causes of lung-related death globally in the near future [2]. Lung cancer, occupational lung disease, atherosclerosis, coronary heart disease, stroke, and other conditions with overlapping etiology are closely related to pulmonary morbidity in COPD [3]. In addition to being crucial for choosing a treatment for COPD, an accurate evaluation of the disease’s severity also affects how complicating conditions like lung cancer are treated. According to recent research, the structural and functional alterations of the lungs that eventually result in obstructive symptoms are caused by the interplay of several potential toxins, environmental factors, and behavioral and genetic variations [4]. Pulmonary function measurement alone cannot adequately grade or predict these. Therefore, cross-sectional imaging is being employed more and more to evaluate the structural and functional lung alterations in COPD, which is primarily characterized by two components: emphysema and airway remodeling. The poor resolution of clinical computed tomography (CT), which is unable to show the majority of the subsegmental airway generations, is the primary cause of this rather arbitrary division [5]. Nevertheless, these factors may coexist and contribute to symptoms to varying degrees in individual patients. Additionally, emphysema that is apparent on CT correlates histopathologically with significant lung tissue destruction [6]. The distinction between an airway-dominant and an emphysema-dominant disease has improved the utility of directing therapy in COPD since airway remodeling is believed to be at least partially reversible, whereas emphysema currently represents irreversible damage [7]. To properly treat patients with emphysema, novel therapeutic regimens, particularly endoscopic lung volume reduction (ELVR) techniques, require a specific workup with high-resolution CT [8].

In recent years, several CT scoring systems that were initially used in acute pancreatitis, such as the CT severity index (CTSI) and the modified CTSI (MCTSI) [9], have been adapted to assess the severity of COPD [10,11]. These scoring systems have proven to be valuable in evaluating the presence and extent of structural lung damage, including emphysema and airway abnormalities. In the case of acute pancreatitis, the MCTSI and CTSI are widely used to evaluate the presence of necrosis and local or extrapancreatic complications [12]. Similarly, in COPD, these CT scoring systems help quantify the extent of lung involvement. The current study aims to compare the efficacy of CTSI and MCTSI to estimate the severity of COPD and its link with clinical outcomes.

## Materials and methods

Research design and selection of the patients

This prospective study was conducted at Lady Reading Hospital, Peshawar, Pakistan, on 60 adult patients who underwent chest CT scans between November 2023 and March 2024 and were identified with COPD according to the criteria set by the Global Initiative for Chronic Obstructive Lung Disease (GOLD). The study was approved by the Institutional Review Board, Lady Reading Hospital, Peshawar, Pakistan, with reference number 270/LRH/MTI. Ethical approval was obtained from the institutional review board, and informed consent was obtained from the participants of the study. Relevant medical history, laboratory test findings, and demographic information were taken. Patients whose imaging suggested other pulmonary conditions, such as chronic bronchitis, interstitial lung disease, or unenhanced CT scan, were excluded. Chest CT scans were conducted after patients presented with symptoms like chronic cough and dyspnea, with imaging typically performed within seven to 14 days of symptom onset (median of 10 days).

Imaging technique

All 60 patients underwent contrast-enhanced chest CT (CECT) using a 40-slice Philips Brilliance CT scan machine. A non-ionic and iodine contrast agent (Iopromide-Ultravist-370) was given by an I.V injection at the dose of 1.5 mL/kg (70-100 mL), with a speed of 3 mL per second after injecting a 20 mL dose of normal saline as a flush. Scans were executed in the porto-venous phase (70 s post-contrast) with the patient in a supine position, covering the area from the lung apices to the diaphragm. Imaging parameters were set at 200 mA/slice and 120 kVp. Axial CT slices were reconstructed with a 3 mm thickness and a collimation of 40 × 0.625 with a pitch of 0.9. These scans assessed lung parenchyma, airway abnormalities, and other pulmonary changes consistent with COPD [13].

Image analysis

Two independent radiologists with 14 and 31 years of experience evaluated all CT images without prior knowledge of the patient’s clinical outcomes. They documented findings related to airway obstruction, emphysematous changes, bronchial wall thickening, and other lung abnormalities indicative of COPD. Both radiologists assigned severity scores using two different CT scoring systems: the Modified Reiff Score for quantifying bronchiectasis severity and a modified version of the GOLD staging based on CT imaging [14]. Discrepancies in scoring between the two radiologists were resolved through consensus.

Clinical outcome parameters

Clinical data, including symptom severity, oxygen dependence, exacerbation history, and mortality, were collected and correlated with imaging findings. Clinical outcomes such as length of hospital stay, frequency of exacerbations, requirement for non-invasive ventilation, ICU admissions, and mortality rates were documented. These clinical parameters were then compared to the CT severity scores based on the GOLD classification: mild, moderate, severe, or very severe COPD. The GOLD classification incorporates spirometry data (FEV1) and was used alongside imaging findings to provide a comprehensive assessment of disease severity [15].

Statistical analysis

IBM SPSS Statistics, version 25.0 (IBM Corp., Armonk, NY), was used to analyze the data. For properly distributed data, continuous variables were displayed as mean ± standard deviation (SD); for skewed distributions, they were displayed as medians (range). Numbers and percentages were used to represent categorical data. Categorical variables were compared using the chi-squared test, with p < 0.05 designated as the statistical significance level. The association between CT severity scores and clinical outcomes, including length of hospital stay, was investigated using Pearson’s correlation coefficient. Based on imaging severity scores, sensitivity, specificity, and predictive values were computed to distinguish between mild and moderate/severe COPD.

## Results

The study population consisted of 60 COPD patients, with a majority being male (40, 66.7%), yielding a male-to-female ratio of 2:1. The mean age was 65.4 ± 8.6 years. Smoking was the most prevalent etiological factor (35, 58.3%), followed by environmental pollutants (15, 25.0%) and occupational exposure (7, 11.7%). Genetic predisposition (alpha-1 antitrypsin deficiency) was found in a smaller subset (3, 5.0%). Emphysema-dominant COPD was observed in 30 (50.0%) patients, while airway remodeling-dominant COPD was present in 25 (41.7%), and five (8.3%) had a combined type. Imaging findings indicated that mild emphysema was more common (18, 30.0%) than severe emphysema (15, 25.0%), with 10 (16.7%) showing bullae formation. No organ failure (OF) was noted in 40 (66.7%) of cases, while 12 (20.0%) experienced single OF, primarily respiratory (10, 16.7%). Most patients had a hospital stay of 0-10 days (34, 56.7%), and 20 (33.3%) required ICU stay. The need for mechanical ventilation was observed in 10 (16.7%) cases, and mortality occurred in five (8.3%) cases (Table 1).

**Table 1:** Demographics, imaging findings, and other data The data has been presented as N (%) and mean ± SD. OF, organ failure

Characteristics	No. of cases (%)
Males	40 (66.7%)
Females	20 (33.3%)
Male-to-female ratio	2:1
Age (mean ± SD years)	65.4 ± 8.6
Etiology	
Smoking	35 (58.3%)
Environmental pollutants	15 (25.0%)
Occupational exposure	7 (11.7%)
Genetic predisposition (alpha-1 antitrypsin deficiency)	3 (5.0%)
Types of COPD	
Emphysema-dominant	30 (50.0%)
Airway remodeling-dominant	25 (41.7%)
Combined	5 (8.3%)
Imaging findings	
Radiologically normal	3 (5.0%)
Mild emphysema	18 (30.0%)
Severe emphysema	15 (25.0%)
Airway thickening	24 (40.0%)
Bullae formation	10 (16.7%)
OF	
No OF	40 (66.7%)
Single OF	12 (20.0%)
Respiratory failure	10 (16.7%)
Cardiovascular failure	2 (3.3%)
Duration of hospital stay (days)	
0-10	34 (56.7%)
10-20	18 (30.0%)
>20	8 (13.3%)
ICU stay	20 (33.3%)
Need for mechanical ventilation	10 (16.7%)
Infections	
Clinical	12 (20.0%)
Radiological	5 (8.3%)
Need for intervention	10 (16.7%)
Mortality	5 (8.3%)

The severity classifications across CTSI, MCTSI, and GOLD varied significantly. MCTSI classified more cases as severe (23, 38.3%) compared to CTSI (15, 25.0%) and GOLD (22, 36.7%). For moderate cases, CTSI assigned 20 (33.3%) as moderate, compared to MCTSI at 15 (25.0%) and GOLD at 14 (23.3%). ANOVA showed significant differences in severity classifications among the three indices (F = 3.5, 4.2, and 5.1; p < 0.05, p < 0.05, and p < 0.01, respectively), indicating that MCTSI identified a higher proportion of severe cases (Table 2).

**Table 2:** Comparison of COPD severity according to CTSI, MCTSI, and GOLD classification ANOVA was used to compare severity grading across CTSI, MCTSI, and GOLD classification. The data has been presented as N (%). CTSI, computed tomography severity index; GOLD, Global Initiative for Chronic Obstructive Lung Disease; ANOVA, analysis of variance

Severity	CTSI (no. of cases, %)	MCTSI (no. of cases, %)	GOLD (no. of cases, %)	Test statistic	p-value
Mild	25 (41.7%)	22 (36.7%)	24 (40.0%)	F = 3.5	<0.05
Moderate	20 (33.3%)	15 (25.0%)	14 (23.3%)	F = 4.2	<0.05
Severe	15 (25.0%)	23 (38.3%)	22 (36.7%)	F = 5.1	<0.01

A significant correlation between airway obstruction levels and OF was observed. In patients with no airway obstruction (n = 30), 28 (93.3%) had no OF, while two (6.7%) had transient OF. Among those with <30% obstruction, six (40.0%) had no OF, two (13.3%) had transient, and seven (47.7%) had persistent OF. For patients with >30% obstruction, three (20.0%) had no OF, one (6.7%) had transient, and 10 (63.3%) had persistent OF. Chi-square analysis showed significant correlations for all obstruction levels, with χ² values of 15.6, 9.3, and 11.2 (p < 0.01), suggesting a direct relationship between increased obstruction and persistent OF (Table 3).

**Table 3:** Correlation of airway obstruction and organ failure The chi-square test was used to analyze the correlation between airway obstruction severity and organ failure. The data has been presented as N (%). The p-value is considered significant at <0.05.

Obstruction level	Organ failure absent	Transient organ failure	Persistent organ failure	Test statistic	p-value
Absent (n = 30)	28 (93.3%)	2 (6.7%)	0	χ² = 15.6	<0.01
<30% obstruction	6 (40.0%)	2 (13.3%)	7 (47.7%)	χ² = 9.3	<0.01
>30% obstruction	3 (20.0%)	1 (6.7%)	10 (63.3%)	χ² = 11.2	<0.01

The median duration of hospital stay increased with severity across both scoring systems. CTSI showed a median of six days for mild, 13 days for moderate, and 15 days for severe cases, with t = 4.8 and p < 0.01. MCTSI indicated 5.5 days for mild, 11.5 for moderate, and 15 for severe cases, with t = 5.0 and p < 0.01. For ICU stay, severe cases (four days) were longer than moderate (two days) and mild cases (0 days), though the difference was not statistically significant. OF rates escalated with severity in both CTSI (χ² = 12.9, p < 0.01) and MCTSI (χ² = 15.4, p < 0.01). Evidence of infection, intervention needs, and mortality also increased significantly with severity (p < 0.05 for all outcomes), indicating that both indices closely reflect clinical outcomes (Table 4).

**Table 4:** Comparison of COPD severity based on CTSI and MCTSI with clinical outcomes T-tests were used for continuous variables; chi-square tests were used for categorical variables to compare severity across clinical outcomes. The data has been presented as N (%). The p-value is considered significant at <0.05. NS, non-significant; COPD, chronic obstructive pulmonary disease; CTSI, computed tomography severity index; MCTSI, modified CTSI

Outcome parameters	CTSI (mild)	CTSI (moderate)	CTSI (severe)	Test statistic	p-value	MCTSI (mild)	MCTSI (moderate)	MCTSI (severe)	Test statistic	p-value
Median hospital stay (days)	6	13	15	t = 4.8	<0.01	5.5	11.5	15	t = 5.0	<0.01
Median ICU stay (days)	0 (n = 0)	4 (n = 8)	4 (n = 11)		NS	0 (n = 0)	2 (n = 1)	4 (n = 18)		NS
Organ failure	1 (3.7%)	12 (63.2%)	12 (85.7%)	χ² = 12.9	<0.01	0	3 (30.0%)	22 (84.6%)	χ² = 15.4	<0.01
Evidence of infection	0	7 (36.8%)	10 (71.4%)	χ² = 9.7	<0.01	0	0	17 (65.4%)	χ² = 11.6	<0.01
Need for intervention	0	4 (21.0%)	11 (78.6%)	χ² = 14.3	<0.01	0	0	15 (57.7%)	χ² = 12.8	<0.01
Mortality	0	0	7 (50.0%)	χ² = 8.2	<0.01	0	0	7 (26.9%)	χ² = 7.4	0.02

CTSI and MCTSI displayed variable concordance with GOLD classification. For CTSI, mild cases had a concordance rate of 25 (100%) with GOLD mild, moderate cases were consistent in eight (40.0%), and severe cases matched GOLD severe in six (40.0%), with χ² values of 10.5, 9.7, and 6.4, respectively (p < 0.05). MCTSI demonstrated higher concordance for severe cases, with mild cases aligning 24 (96.0%) with GOLD mild, moderate in seven (35.0%), and severe in eight (53.3%), yielding χ² values of 11.1, 8.8, and 6.9, respectively (p < 0.05). Overall, MCTSI showed better alignment with GOLD severity, particularly in severe classifications (Table 5).

**Table 5:** Consistency of CTSI and MCTSI with GOLD classification of COPD The chi-square test was used to assess the consistency of CTSI and MCTSI with GOLD classification. The data has been presented as N (%). The p-value is considered significant at <0.05. COPD, chronic obstructive pulmonary disease; CTSI, computed tomography severity index; MCTSI, modified CTSI; GOLD, Global Initiative for Chronic Obstructive Lung Disease

Consistency of scoring	GOLD (mild, n = 25)	GOLD (moderate, n = 20)	GOLD (severe, n = 15)	Test statistic	p-value
CTSI mild	25 (100%)	1 (5.0%)	0	χ² = 10.5	<0.01
CTSI moderate	0	8 (40.0%)	9 (45.0%)	χ² = 9.7	<0.01
CTSI severe	0	11 (55.0%)	6 (40.0%)	χ² = 6.4	<0.05
MCTSI mild	24 (96.0%)	2 (10.0%)	0	χ² = 11.1	<0.01
MCTSI moderate	1 (4.0%)	7 (35.0%)	7 (46.7%)	χ² = 8.8	<0.01
MCTSI severe	0	11 (55.0%)	8 (53.3%)	χ² = 6.9	<0.05

CTSI and MCTSI both showed high sensitivity and specificity when compared with the GOLD classification for diagnosing moderate to severe COPD. CTSI demonstrated a sensitivity of 91.4% and specificity of 96.0% (χ² = 12.3, p < 0.01), with 32 true positives (91.4%), one false positive (5.0%), three false negatives (8.6%), and 24 true negatives (96.0%). MCTSI had a sensitivity of 94.4% and specificity of 92.0% (χ² = 11.9, p < 0.01), with 34 true positives (94.4%), two false positives (10.0%), two false negatives (5.6%), and 23 true negatives (92.0%). These results indicate that MCTSI is slightly more sensitive, while CTSI is marginally more specific in identifying moderate to severe COPD cases (Table 6).

**Table 6:** Sensitivity and specificity of CTSI and MCTSI for diagnosing moderate/severe COPD (using GOLD as reference) The chi-square test was used to determine the sensitivity and specificity of CTSI and MCTSI, with GOLD classification as the reference standard. The data has been presented as N (%). The p-value is considered significant at <0.05. TP, true positive; FP, false positive; TN, true negative; FN, false negative; COPD, chronic obstructive pulmonary disease; CTSI, computed tomography severity index; MCTSI, modified CTSI; GOLD, Global Initiative for Chronic Obstructive Lung Disease

Scoring method	Moderate + severe (TP + FP)	Mild (TN)	Sensitivity	Specificity	Test statistic	p-value
CTSI	32 (91.4%) TP, 1 (5.0%) FP	3 (8.6%) FN, 24 (96.0%) TN	91.4%	96.0%	χ² = 12.3	<0.01
MCTSI	34 (94.4%) TP, 2 (10.0%) FP	2 (5.6%) FN, 23 (92.0%) TN	94.4%	92.0%	χ² = 11.9	<0.01

## Discussion

The purpose of this study was to evaluate the degree of severity of the COPD with the help of the CTSI and MCTSI and to link these scores with clinical outcomes based on the GOLD 2012 classification. Our results demonstrated a strong correlation between both CT-based scoring systems and key clinical outcomes, including mortality, mean hospital stay, persistent OF, signs of infection, and the requirement for clinical intervention.

Both CTSI and MCTSI showed high interobserver agreement among the two radiologists, with no variation in scoring, guaranteeing consistency. Furthermore, there was good consistency between both CT grading systems and severity classification according to the GOLD criteria. This reflects that both scoring systems are reliable in the assessment of COPD severity. CTSI was slightly less sensitive but 100% specific in categorizing moderate or severe cases, while MCTSI exhibited 100% sensitivity, effectively capturing every severe case. However, there was no statistically significant difference between CTSI and MCTSI in terms of overall severity rating.

A notable distinction between MCTSI and CTSI lies in the inclusion of extrathoracic issues such as pleural effusion, ascites, and vascular complications in the MCTSI. In our study, 55% of patients had such complications, leading to higher MCTSI scores by 2 points on average compared to CTSI. This ability to account for extrathoracic manifestations makes MCTSI particularly useful in assessing COPD patients with complex presentations. Additionally, the MCTSI simplifies the classification of emphysema by grading it as either <30% or >30%, which is more straightforward compared to the more granular approach of CTSI.

Previous studies have similarly demonstrated a strong association between CT severity scores and clinical outcomes in COPD, including length of hospital stay, ICU admission, persistent OF, infection, and mortality [16,17]. In our study, both types of CT scans were significantly correlated with these clinical parameters, except for the length of ICU stay, which did not show a strong association. The fact that just 19 out of 60 patients needed ICU admission may be the cause of this. Nevertheless, the overall clinical severity, as reflected in both CT scores, showed strong predictive value for adverse outcomes, including persistent OF and mortality.

Importantly, both types of CT scans showed good consistency with severity scores according to the GOLD criteria, with MCTSI performing marginally better in classifying severe cases. These findings align with those from previous studies, such as Raghuwanshi et al., who also found that MCTSI tends to be more sensitive than CTSI in identifying high-risk cases of acute pancreatitis [18]. Our data further support this, as MCTSI accurately classified all patients requiring clinical intervention or experiencing complications like infections, while CTSI underestimated the severity in a subset of patients.

We also found a strong association between emphysema severity (>30%) and the development of persistent OF, consistent with the literature. This relationship between higher emphysema burden and organ dysfunction has been well-documented in previous studies, such as those conducted by Verma et al., which demonstrated a direct link between the extent of necrosis (analogous to tissue destruction in COPD) and poor clinical outcomes [19]. In our study, patients with persistent OF were at significantly higher risk of mortality, particularly when infection was present.

The values for the sensitivity and specificity of modified CTSI in classifying COPD cases as mild or moderate to severe were 100% and 92.3%, respectively. In comparison, CTSI had a sensitivity of 97.1% and a specificity of 100%. While MCTSI tended to overestimate the severity in a few mild cases, it was more effective in identifying high-risk patients who required closer clinical monitoring and intervention. This makes MCTSI a valuable tool in clinical settings where early identification of complicated COPD is critical for guiding treatment decisions [20].

A small subset of patients (15.4%) had CT findings suggestive of severe disease, with >30% emphysema and other extrathoracic complications, yet had a relatively mild clinical progression. The participants, predominantly young males with long-term smoking histories, did not exhibit OF or infections, suggesting that CT findings alone may not always align with clinical outcomes. More extensive research is required to validate these observations and explore whether certain phenotypes of COPD may have better clinical outcomes than predicted by imaging alone.

There were some limitations to our study. Our population was biased toward more severe cases of COPD, as patients with mild disease often did not undergo CT imaging, consistent with clinical practice guidelines that recommend limited imaging for milder cases. Furthermore, our study lacked clinical scoring techniques such as the BODE Index (body mass index, airflow obstruction, dyspnea, and exercise capacity), which would have shed more light on the connection between CT findings and clinical severity. The fact that the CTSI and MCTSI scores were taken at the same interpretation session was another drawback. This could have created bias because the radiologist who assigned one score knew about the other.

## Conclusions

Both CTSI and MCTSI are useful tools for evaluating the severity of COPD, with MCTSI offering an advantage in sensitivity, particularly for identifying severe cases and extrathoracic complications. While both scoring systems are effective in predicting clinical outcomes, MCTSI’s enhanced ability to capture complex disease presentations makes it a valuable tool for the early detection of high-risk patients. Future studies should aim to validate these findings in larger cohorts and further explore the integration of CT scoring with clinical indices for comprehensive COPD management.
